# Irrational use of antibiotics in Iran from the perspective of complex adaptive systems: redefining the challenge

**DOI:** 10.1186/s12889-021-10619-w

**Published:** 2021-04-23

**Authors:** Zahra Sharif, Farzad Peiravian, Jamshid Salamzadeh, Nastaran Keshavarz Mohammadi, Ammar Jalalimanesh

**Affiliations:** 1 School of Pharmacy, Shahid Beheshti University of Medical Sciences, Tehran, Iran; 2 Health Promotion School of Public Health, Shahid Beheshti University of Medical Sciences, Tehran, Iran; 3 Scientific Committee of UNESCO global Chair on Health and Education Associate Editor, Health Promotion International, UZH, Zurich, Switzerland; 4 Iranian Research Institute for Information Science and Technology (IRANDOC), Tehran, Iran

**Keywords:** Complex adaptive systems (CAS), Complexity sciences, Rational use of antibiotics, Pharmaceutical policy

## Abstract

**Background:**

Irrational use of antibiotics is proving to be a major concern to the health systems globally. This results in antibiotics resistance and increases health care costs. In Iran, despite many years of research, appreciable efforts, and policymaking to avoid irrational use of antibiotics, yet indicators show suboptimal use of antibiotics, pointing to an urgent need for adopting alternative approaches to further understand the problem and to offer new solutions. Applying the Complex Adaptive Systems (CAS) theory, to explore and research health systems and their challenges has become popular. Therefore, this study aimed to better understand the complexity of the irrational use of antibiotics in Iran and to propose potential solutions.

**Method:**

This research utilized a CAS observatory tool to qualitatively collect and analyse data. Twenty interviews and two Focus Group discussions were conducted. The data was enriched with policy document reviews to fully understand the system. MAXQDA software was used to organize and analyze the data.

**Result:**

We could identify several diverse and heterogeneous, yet highly interdependent agents operating at different levels in the antibiotics use system in Iran. The network structure and its adaptive emergent behavior, information flow, governing rules, feedback and values of the system, and the way they interact were identified. The findings described antibiotics use as emergent behavior that is formed by an interplay of many factors and agents over time. According to this study, insufficient and ineffective interaction and information flow regarding antibiotics between agents are among key causes of irrational antibiotics use in Iran. Results showed that effective rules to minimize irrational use of antibiotics are missing or can be easily disobeyed. The gaps and weaknesses of the system which need redesigning or modification were recognized as well.

**Conclusion:**

The study suggests re-engineering the system by implementing several system-level changes including establishing strong, timely, and effective interactions between identified stakeholders, which facilitate information flow and provision of on-time feedback, and create win-win rules in a participatory manner with stakeholders and the distributed control system.

## Background

Irrational use of medicines poses a formidable challenge to health systems in many countries [1–4]. The practice includes over-or under-prescription, inappropriate self-medication, polypharmacy, incomplete course of medications, overuse of antibiotics and injectable drugs, and non-adherence to clinical guidelines [3, 5, 6]. The World Health Organization (WHO) has ominously warned that still “more than half of all medicines are prescribed, dispensed or sold inappropriately, and half of all patients fail to take medicines prescribed to them correctly” [7]. The far-reaching consequences of irrational use of antibiotics can range from increased morbidity and mortality, enhanced medication errors, increased healthcare costs, patients’ mistrust in physicians and healthcare authorities [7–9]. Among all medicine categories, irrational use of antibiotics has brought about additional undesirable health outcomes like antibiotic resistance [10–12] and has remained as a substantial public health problem globally, not least in developing countries [13, 14].

Despite all appreciable efforts to promote the rational use of antibiotics in Iran such as establishing the National Committee on Rational Use of Drugs (NCRUD), codifying some regulations and guidelines, educating the public on the rational use of antibiotics, enforcing new pharmaceutical protocols in hospitals to rationalize the use of most expensive antibiotics, and conducting several research studies in this field, indicators continue to show a suboptimal prescription and use of antibiotics in Iran [15, 16]. For example, the number of prescriptions including antibiotics is still above the national and international standards [17]. Physicians prescribe antibiotics irrationally [18], and although antibiotics are not classified as over-the-counter drugs, pharmacies usually provide antibiotics to consumers even without prescription unlawfully [15]. Because of insufficient public knowledge about antibiotics, patients’ adherence to antibiotics therapy is low, and self-medication with antibiotics is common among Iranians [15, 19, 20]. As a result, Antimicrobial Resistance (AMR) rate is increasing [21]. Studies that have explored the current situation of irrational use of antibiotics in Iran, pointed out the insufficiency of previous efforts in improving the status quo and, subsequently, the necessity of a shift in policymaking and defining the problem.

Recently, providing healthcare services has shifted from a single prescriber and user in a single setting to multiple providers and users at different levels of settings, presenting healthcare systems with many unexpected complexities and challenges [22]. In fact, like most health problems, irrational use of antibiotics often stems from its multi-disciplinary and complex nature, so it should not be reduced to linear root causes [23]. Generally, classic approaches to solving health problems are inadequate as they often tend to ignore the subtle inherent complexities of the problem in favor of its easily visible features [24]. Therefore, in the face of such baffling complexities, achieving public health goal would definitely require going beyond overly simplistic notions [25].

Health systems can be a classic embodiment of a complex system as they involve many autonomous but connected and even dependent actors at different settings, changing is continuous, many feedback loops are running, and the effect of a single intervention in one part may be observed in other parts of the system [26]. Recently complexity science approach to health, settings, and organizations has emerged as a promising approach that can provide a more thorough understanding of complex health issues [27–32]. Similarly, applying the Complex Adaptive Systems (CAS) theory, a derivative of complexity science, to understand, explore, scale-up, evaluate and research in health systems has become popular among researchers in recent years [25, 33–36].

## CAS characteristics

### A multi-agent system with many interactions

Diverse interactive agents are a very substantial part of healthcare systems with power in shaping the system, its behavior, and making decisions [26, 37]. These agents, considered as systems in their turn, can be a part of other systems [27, 38]. As a result, agents are interacting in a nested, multilevel, and networked system. The interactions between agents in CAS are commonly non-linear [30].

### A dynamic system with ever-adapting and learning agents

Agents and the whole system alter their behaviors and interactions according to the characteristics of other agents and the impact of external circumstances, in other words, they adapt to new conditions [25, 39, 40]. The interactions between multiple agents, feedback mechanisms, and flow of information are key learning sources and drivers of adaptation which is an essential phenomenon in CAS [27]. Continuous adaptation and intermittent changes cause systems to be more dynamic than static.

### De-central control of the agents

Agents’ autonomous behavior, non-linear interactions, and dynamic nature make the central and full control of the system impossible [41]. The outputs of CASs are more yielded from a process of self-organization rather than hierarchical external control [27]. It can be very common for CASs to expose little response to many controlling efforts, or to show a big change in response to a tipping point [30].

### A system with emergence behavior

One of the pivotal characteristics that define a CAS is emergence [36]. The core concept of this feature is that the whole behavior of the system is probably greater and more complex than the sum of individual behaviors [25, 40]. The interplay of elements results in behavior in the whole system, which cannot be easily observed and predicted [27, 42, 43].

Although healthcare systems have been acknowledged as CAS by many scientists [26, 29, 30, 32, 39, 42, 44], it seems that it has rarely been translated into practice. Considering a paradigm shift toward a description of healthcare systems as complex systems in recent years and the insufficiency of the traditional approaches, it is, therefore, worthwhile to explore the premises of complexity science to approach health care problems. There has been little exploration of the potential benefits of the application of CAS to better analyze and provide policy and practical recommendations for the irrational use of antibiotics. Drawing upon the CAS observatory tool, this study aims to observe and prescribe the problem in the healthcare system of Iran through the lens of CAS, and apply the CAS explanatory capability to explain, understand and refine the issue and the system emergent behaviors to provide complexity-informed recommendations for policy and practice.

## Methods

The study was designed as qualitative research utilizing qualitative data collection and analysis methods. Qualitative methods are suitable to explore and explain complex social phenomena as well as social complex systems [45]. They can help us understand social complex issues and address processes that arise over time. Complex Adaptive System theory was utilized to guide the formulation of research questions, data collection, and analysis. The study used the CAS observatory and explanatory tool [27], which consists of the following five items which formed the main themes of qualitative analysis:

1. Identification of agents, interactions, and system structure
2. Description of the information flow in the system
3. Distinction of feedback loops
4. Recognition of rules and values that run in the system
5. Exploration of adaptation, self-organized and emergent behavior of agents

This study conducted two qualitative data collection methodologies: (I) semi-structured interview to collect data from individuals and (II) Focus Group Discussion (FGD) designed to discuss the use of antibiotics with those whom we assumed might express their opinions in a group discussion more openly and comfortably. In order to fully understand the system, the data was enriched with policy document reviews. According to the CAS observatory tool, the probing questions for interviews and FGD were developed [see supplementary file]. Additionally, interviewees were free to state additional viewpoints on the topic so that we could get more in-depth views of the participants. In order to find key stakeholders, available literature related to antibiotics use, rational use of drugs, and antimicrobial resistance were reviewed. All relevant studies published by September 2017 were included. The review included grey literature as well as peer-reviewed papers and the results were validated by policymakers. We identified five key agent types at different levels of antibiotics use in Iran as follows: 1. Policymakers, 2. Prescribers, 3. Producers and distributors, 4. Trainers and researchers, 5. Patients or consumers. The first four levels were chosen to be interviewed and the last, i.e. patients or consumers, were assigned to FGDs. This categorization provided a holistic understanding of the issue and captured previously unmet and unexplored aspects of the rational use of antibiotics.

Ethical approval was obtained from the Institutional Research Ethics Committee School of Pharmacy and Nursing & Midwifery – Shahid Beheshti University of Medical Sciences. All interviewees provided verbal informed consent to participate and audiotape of interview or FGD.

Altogether, twenty interviews and two FGDs were conducted. The participants were purposefully selected and entered the study. Data collection was carried out from December 2017 to August 2018. All transcriptions of both interviews and FGDs supplied rich material for analysis. Qualitative analysis of the data was facilitated through MAXQDA 2018 software. The core notions of the CAS observatory framework, as introduced earlier, constituted the main concepts of analysis (thematic analysis). The validity of coding was improved through analyst triangulation, three researchers cross-coded the raw material and discussed the analysis at regular intervals.

## Results

We first present an overview of the irrational use of antibiotics in Iran and, followed by describing the system as a CAS using the CAS observatory tool.

### Confirming the irrational use of antibiotics in Iran

Generally, the irrational use of antibiotics in Iran was acknowledged by almost all interviewees, although some argued that some progress had been achieved in recent years. Many participants estimated that patients’ adherence to antibiotic therapy was medium to low, as they did not follow the instructions properly and did not complete the antibiotics therapy course. Antibiotics self-medication was identified to be prevalent especially when patients suffered from the common cold, Self-medication was reported in nearly 40% of respiratory disease and common cold cases [19]. They also reported that many pharmacies deliver antibiotics to customers without prescription and therefore antibiotics were easily accessible to the public. It was identified that, for a variety of reasons such as patients’ insistence and satisfaction, physicians usually prescribed antibiotics irrespective of medical guidelines or laboratory tests. These factors sometimes led to inappropriate dosing, improper combination therapy, and irrational prescription of injectable antibiotics. All these behaviors have ultimately culminated in a high incidence of antibiotic resistance. For example, between 2013 and 2014, about half percent of *Escherichia coli* microorganisms in Iran were resistant to third-generation cephalosporins and fluoroquinolones [21].

### Considering antibiotics use system as a CAS

#### Agents and their interactions

We identified several diverse and heterogeneous agents (stakeholders) regarding the use of antibiotics in Iran, organized in subsystems or groups of medical universities, hospitals, patients, etc. Moreover, some interviewees suggested that significant diversity could be seen in similar agents. For instance, the perceptions and behavior of physicians, pharmacists, and patients regarding antibiotics are not always similar as they stem from a different level of knowledge, attitude, experiences, and individual and organizational governing values and rules.

All these agents can fall into two major categories: supply-oriented agents and demand-oriented agents. Supply-oriented agents refer to all agents that can provide antibiotics to end-users (can be patients or not) or play a role in the process of supplying, for instance by regulating the procedures or training physicians. Demand-oriented agents, on the other hand, refer to all agents that are prone to use antibiotics, both prescribed and unprescribed, or, like the media, can alter the behavior of antibiotics use on either side. It should also be noted that some agents can be shared members of both categories as they can affect both the supply and demand sides of antibiotics use. These agents include the Ministry of Health (MOH), Iran Food and Drug Administration (IFDA), prescribers, and hospitals. Within these arrangements, agents can be characterized by their properties, roles, importance or power, and objectives [see Table 1 file].

**Table 1:** Agents, their properties, role, power, and objectives

Agents or subsystems	Role	Related components	Importance/power	Objectives/ incentives	
Supply-oriented	Ministry of health and medical education (MOH)	It is the main policy-making and stewardship agent that decides about macro health policies. It also regulates and finances all service provisions in healthcare	Deputy of curative affairs, deputy of education and deputy for health. Some components like medical universities existing all provinces all around the country, are defined as a major and separate agent.	The most important governmental agent that oversees all the actions and processes in health care system and is in charge of all things related to public health.	Providing health and hygiene to all citizens.
	Iran Food And Drug Administration (IFDA)	Supervising and regulating all the processes of manufacturing, distributing, and use of antibiotics. It is authorized and financed by MOH	The National Committee on Rational Use of Drugs (NCRUD), General directorate of Pharmaceutical and narcotic affairs	It is the most substantial supervisory body that directly controls all the processes of supply and access to antibiotics. It makes policies related to access to antibiotics and compiles pharmaceutical guidelines through collaborating with other related bodies. It is also responsible for the rational use of all drugs, especially antibiotics.	Enabling access to effective and safe medicines in a rational way.
	Basic health Insurance companies	They reimburse antibiotic drugs.	There are three major basic public insurance companies in Iran. Besides National health Insurance called Iran Health Insurance Organization (IHIO) and Social Security Insurance (SSI), there is also Ministry of Defense Health Insurance Organization. There are also many private insurance companies, but they are not usually considered as major players	They are the main enforcing levers of health policies in Iran. They can rationalize the use of antibiotics by developing limitations and special regulations for reimbursing antibiotics.	Trying to minimize their cost.
	Medical universities	They are authorized by MOH. They educate and train physicians, pharmacists, specialists, and other healthcare professionals who can order or deliver antibiotics.	Vice chancellor of Food and Drug administration in medical universities, Educational hospitals and pharmacies, professors, students and medical residents	The power of medical universities is pretty high because MOH and IFDA enforce their supervision of prescribing and delivering antibiotics through Food and Drug Departments of different medical universities. All departments have a RUD committee. They periodically check upon physicians under their supervision to ensure their acceptable prescription practices and provide feedback to them. Additionally, they play a critical role in promoting physicians and pharmacists’ knowledge and practice of antibiotics by continuing medical education (CME) programs. They can also supervise all promotional activities of pharmaceutical companies in hospitals, pharmacies and clinics, etc.	Training qualified and knowledgeable physicians, pharmacists and other health care professional. Helping health care professionals maintain competence and learn about new and developing areas of their field.
	Research centers	Conducting clinical and non-clinical researches related to the rational use of antibiotics.	–	The outcome of their researches may influence two main processes: policy-making and antibiotic prescription.	Communicating and collaborating with policy-makers and prescribers adequately. Carrying out feasible and practical researches.
	Islamic Republic of Iran Medical Council (IRIMC)	IRIMC is the largest national non-governmental organization in which all health care professionals (except nurses) have to register to be granted permission to practice in the country. It regulates health care professionals’ collaborations with other associations. It has developed many regulations and guidelines related to medical practice standards.	It has more than 190 branches all over the country in different cities.	It can play an important supervisory role in physicians and pharmacists’ practices. Additionally, it can affect the physicians’ prescription behaviors through their contribution to the development and enforcement of guidelines as well as educational programs.	Improving and modifying medical affairs in Iran. Supporting patients’ and health care professionals’ rights. Promoting medical knowledge in Iran.
	Scientific and guild NGOs	Providing educational and research services. They support healthcare professionals’ rights.	Many scientific and non-scientific NGOs are practicing in different fields of medical sciences.	They play a significant role in other health care professionals’ behavior such as nurses, dentists, etc. They also enforce regulations, influence antibiotic prescription practices, and make connections between different groups of healthcare professionals.	Improving education, training and research services. Protecting physicians and pharmacists’ monetary and non-monetary rights.
	Pharmaceutical companies	Producing or importing necessary pharmaceuticals of the country. Introducing, providing and promoting antibiotics to prescribers and pharmacies through diverse promotional activities.	It includes many manufacturing and importing companies.	They can highly affect both demand and especially supply sides of the antibiotic market. They promote their products through different ways such as giving free samples, discounts, gifts, etc.	Making more profit through grabbing and maintaining more market share.
	Prescribers	They prescribe antibiotics to the patients.	It includes physicians (General Practitioners or specialists), dentists and midwives.	After MOH, they are the second important agent in both supply- and demand-oriented group agents. They determine the number and quality of antibiotic prescription.	A wide range of interests from enhancing rational use of antibiotics and patients’ quality of life to monetary objectives and visiting more patients.
	Hospitals	Providing in-patient care service and also most of the time, out-patients’ services.	Physicians, nurses, clinical pharmacists, Pharm-D, pharmacotherapy committee, and antibiotics stewardship committee	Their practice highly affects the volume and the quality use of a wide spectrum of injectable antibiotics.	Controlling antibiotic use, improving the rational use of antibiotics in order to prevent antimicrobial resistance at hospitals
	pharmacists	Delivering antibiotics to patients and to the general public. They also have to explain and give consultation to patients about the use of antibiotics in terms of how to use, interactions and side effects, etc.	–	Irrespective of codes of action, they sometimes provide antibiotics to the public and patients over the counter. They occasionally collaborate with pharmaceutical companies to sell more antibiotics. They can also collaborate with physicians to prescribe more antibiotics.	Providing good service delivery and maximizing their profit.
Demand-oriented	mass media	Improving public knowledge about antibiotics through educational programs and contents.	TV, social networks like Instagram, telegram, Facebook, etc.	They can highly affect public knowledge, attitudes about antibiotics. They also help to modify the general public’s life style and alter their perception of antibiotics, physicians and pharmacists.	To attract and maintain more audiences.
	Patients and public	use antibiotics (final consumer)	Patients, patients’ families and friends, public population	They are the most important agent on the demand-side. Their health literacy, knowledge, perceptions, expectations and experiences highly affect antibiotic use.	Living better and more comfortably. Having the best treatment in the world for their illnesses. Having the lowest cost services.

**Fig. 1:**
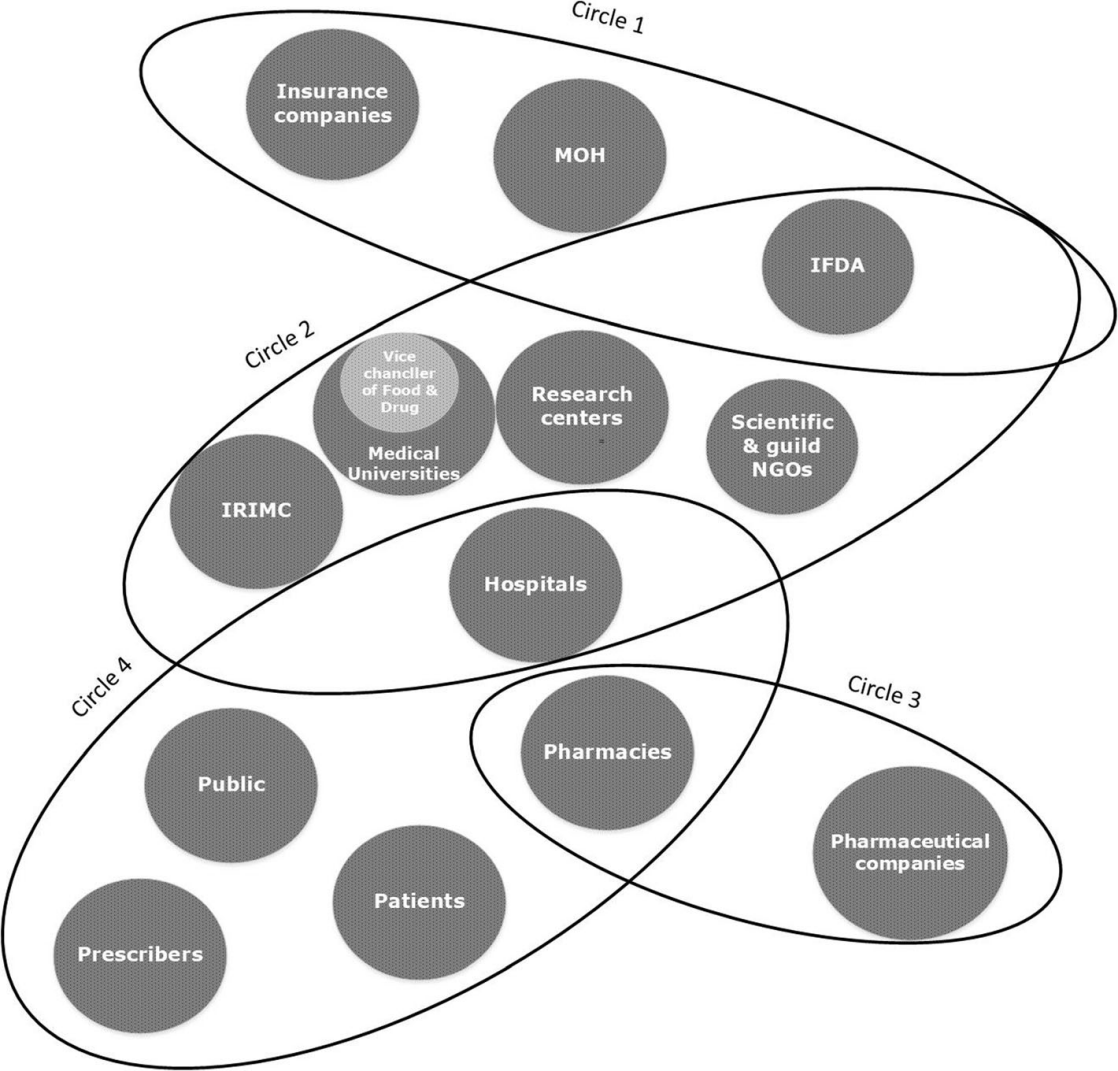
Subsystems of antibiotics use in Iran Circle 1 represents agents who make policies, regulate the system and reimburse antibiotics. Circle 2 represents monitoring agents that investigate the implementation of regulations or provide evidence to make policies. Circle 3 points at agents which produce and dispense antibiotics and circle 4 represents agents who contribute to the core function of antibiotic prescription and use

After the original mapping of agents, we could break down the whole system into four interdependent layers as subsystems (Fig. 1). Circle 1 comprises MOH, IFDA, and insurance companies that make policies, regulate the system, reimburse and provide access to antibiotics. Circle 2 consists of those agents who monitor the implementation of regulations and guidelines, help circle 1 to enforce the regulation and make better policies by providing the necessary evidence. IFDA is present in both circle 1 and circle 2 because it contributes to both policymaking and supervision. Circle 3 includes pharmaceutical companies and pharmacies that produce and distribute antibiotics. However, the core functions of supply and use of antibiotics take place in circle 4. Physicians include general practitioners and specialists, patients, the public, pharmacies and, hospitals are overlaid by this circle. Pharmacies are distributing antibiotics and ensure access to them, therefore, they are placed in both circle 3 and circle 4. Hospitals are potentially very important agents that produce information and pieces of evidence needed for decision makings. Therefore, they are present in both circle 2 and circle 4. Although these circles are operating at different levels and can be broken up, they are highly interdependent with overlaps. Most participants argued that most of the current solutions to tackle the irrational use of antibiotics in Iran have failed to adequately take into account all agents, their role, and power.

#### Nested structured interaction

**Fig. 2:**
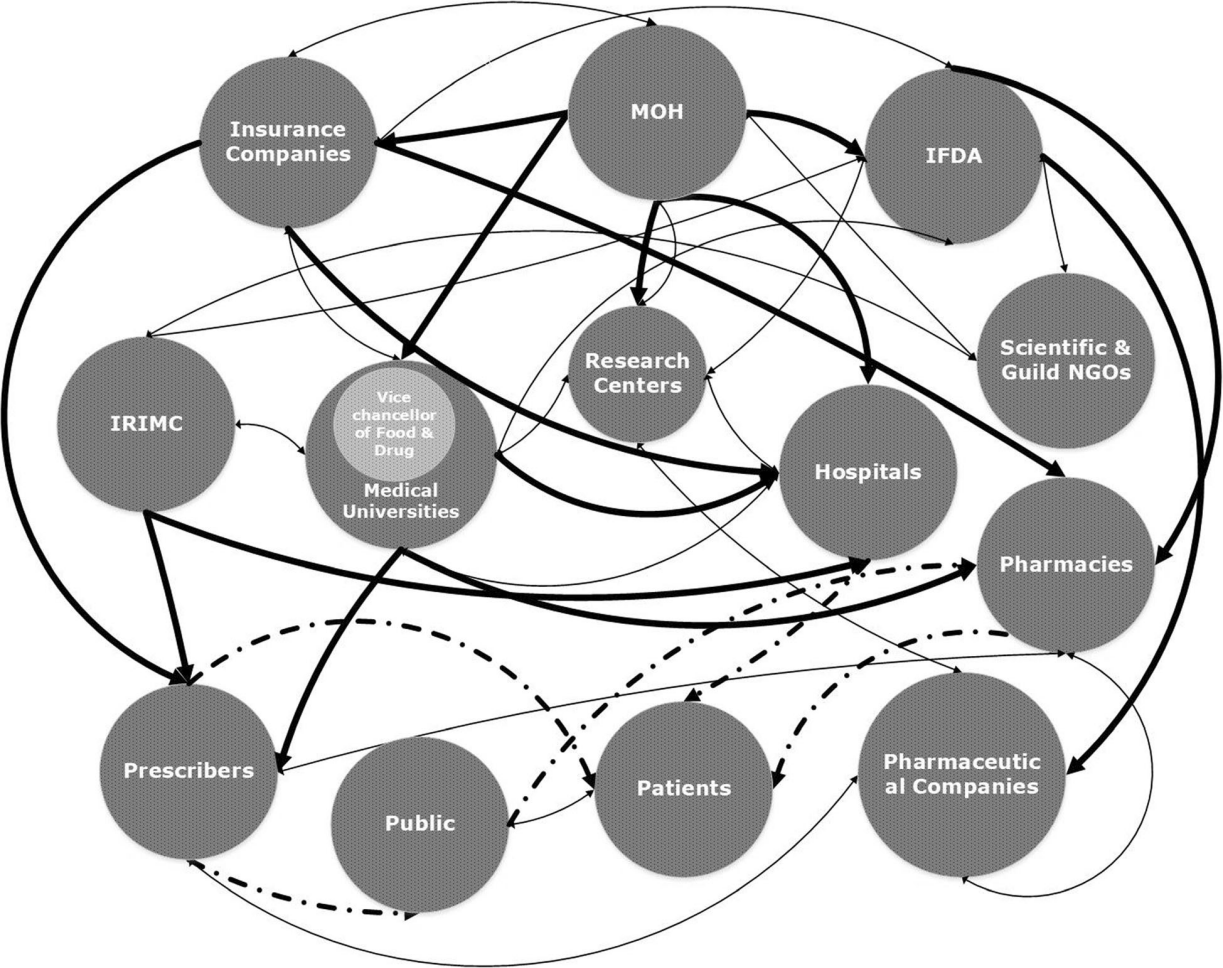
Main agents’ interactions in antibiotics use in Iran and their contribution to the formation of a networked interaction structure Thick lines represent type-one interactions between agents that are governance-oriented and rule-based. Dots and dashes display type-two interactions, where the heart of antibiotics use lies. Lastly, thin lines illustrate type-three interactions between agents, which are reciprocal

Data analysis showed that there are diverse and several interactions and interaction patterns between agents (stakeholders) in antibiotics use in Iran, leading to a nested structure and also a network system. These interactions were identified to be influential in decision making and behaviors regarding prescription or use of antibiotics, based on rules enforced or information exchanged through these interactions. Figure 2 shows the contribution of agents’ interactions to the formation of a nested and multilevel system.

We identified three patterns of interactions between agents. Type one represents governance and supervisory-oriented top-down interactions that regulate the activities, supervise the procedures, and finance or reimburse medical costs. For example, there can be several formal governance-oriented and so rule-based interactions of MOH with IFDA, top-down interactions of medical universities with hospitals, or financial and legal interactions of insurance companies with physicians and pharmacies regarding the reimbursement of service provision and medical costs. Type two represents interactions relates to service provision, where the main action of antibiotics use occurs. Several informal and formal interactions between physicians, pharmacists, patients, and hospitals are subsumed under this rather broad category. Finally, type three embodies reciprocal interactions, where agents have interactions based on professional collaboration or contract-based corporation. For instance, there are collaborative interactions between MOH and IFDA with medical universities and research centers, pharmaceutical companies contract research centers to conduct their clinical trial researches, IFDA has interactions with scientific Non-Governmental Organizations (NGOs), Islamic Republic of Iran Medical Council (IRIMC) and academia. Pharmaceutical companies have many formal and also informal interactions with physicians and pharmacies. Through all these types of interactions, information and financial resources get exchanged, regulations are enforced and antibiotics are delivered to patients or the general public as a whole. For example, insurance companies send prescription information that IFDA, MOH or medical universities have requested, hospitals can provide data and information which research centers and medical universities need for their researches. As shown in Fig. 2, almost all interactions of circle 1 and circle 2 with circle 3 and circle 4, can be described as top-down governance-based relationships, while interactions between circle 1 and circle 2 can be described as a mixture of governance-based and reciprocal relationships. Circle 3 and circle 4 exhibit reciprocal interactions.

Many participants believed that interactions between key stakeholders in the antibiotics use system in Iran were often insufficient, ineffective, or non-systematic. For example, interviewees made a point of highlighting the discontinuous and insufficient reciprocal interactions between research centers and policy-making entities like MOH, IFDA, insurance companies, and research centers. Inadequate interactions of IFDA with other agents such as IRIMC were mentioned by the majority of participants. Lack of adequate supervision on prescribing and delivering antibiotics, incomplete implementation of programs, and improper enforcement of rules and regulations were identified as the consequences of current ineffective interactions and inadequate coordination between different parts of the MOH and the whole system of antibiotics use in Iran.

### Information flow

Almost all respondents were dissatisfied with the current information flow of the system regarding antibiotics. They argued that vital information circulated inefficiently and inadequately or with delay in the systems which adversely affect antibiotics use. For example, insurance companies produce antibiotics prescription information by analyzing physicians’ prescriptions and send this information to IFDA or MOH, but there have been some logistic problems in IFDA recently that have impeded data transformation. The infrastructure of Information Technology in IFDA did not have enough capacity to integrate and process prescription-related information to be used effectively for policymaking or providing feedback. Therefore the information on antibiotics use such as the percentage of prescriptions containing antibiotics is not updated to show a clear picture of the current situation. Other important sources of antibiotics use data are hospitals and departments of the Food and Drug Administration in medical universities. They should report Rational Use of Drug (RUD) indicators such as the mean item per prescription and the percentage of prescriptions containing MOH and IFDA, but they do not do it regularly and accurately. As a result, MOH and IFDA do not have on time, accurate and detailed information of antibiotics prescription. In the absence of correct and updated information on antibiotics use, any planning and policymaking to rationalize prescription and consumption would be challenging. Many patient participants in FGDs complained about the incomplete information about antibiotics use imparted to them by physicians and pharmacists. Physicians and pharmacists are supposed to educate their patients about antibiotics administration and use, but often they fail to do so. This may consequently lead to irrational use of antibiotics, such as not completing treatment course and discontinue antibiotics use while they are recovering. Respondents working for research centers or scientific NGOs stated that despite their willingness to collaborate with IFDA or MOH to conduct some useful research and participate in healthcare decision-making, the stage had not been set for the smooth flow of necessary information.

Moreover, many participants criticized the quality and accuracy of information about antibiotics production and distribution run in the system. Pharmaceutical companies are supposed to send their data on medicine production and distribution (i.e. the number of antibiotics distributed to pharmacies) to IFDA. However, distributing companies often send their information with delay and even the accuracy of the data is always seriously in question. Based on the information provided by pharmaceutical distributing companies, IFDA should publish a report on official pharmaceutical statistics annually. As a result of the delay in receiving the questionable information, this report usually is published with a delay and its information is not reliable. Moreover, some interviewees argued that information circulation also happens through informal and non-documented channels, and so much of officially documented information exchange slips unnoticed. For example, through personal relationships between some employees in IFDA and pharmaceutical companies, the information on distributed antibiotics can be transmitted timely but not through formal and official channels. However, this non-official information flow is temporary and lasts only as long as those people are in charge of IFDA.

### Feedback loops

The main identified formal feedback mechanism in the system of antibiotics use in Iran is RUD indicators such as the mean item per prescription and the percentage of prescriptions containing antibiotics produced by insurance companies. Analyzing this information, regulatory bodies evaluate their programs and the effectiveness of their practices toward the rational use of antibiotics. In some cases, they send feedback reports to physicians who have not met prescription indicators and ask them to adhere to guidelines and regulations. However, no systematic feedback mechanisms were identified from MOH, IFDA, or insurance companies to prescribers and other practitioners or vice versa in the system.

There is some informal and even not easily observable feedback that forms the behavior of the system as follows:

Patients’ experience of antibiotic resistance which, increases the treatment failure of infectious diseases was found to negatively influence patients’ adherence to the treatment course, thus, encouraging them to discontinue antibiotic therapy or consume more potent antibiotics. It was found to contribute to some physicians’ reluctance to adhere to medical guidelines, which can, in turn, increase irrational antibiotics prescription and perpetuate the vicious circle of antibiotic resistance.

Patients’ experience with previously used antibiotics may also affect the patient-doctor relationship and their trust in physicians. Many respondents said that in many cases physicians prescribe antibiotics irrationally because patients do not trust them and insist on receiving newer or more antibiotics. Physicians receive feedback about the success of their prescription from their patients by observing their symptoms. In addition, if patients are not satisfied with the dose or type of prescribed antibiotics, they may change their physicians or buy antibiotics from a pharmacy without a prescription. Considering that there are direct financial relationships between patients and physicians in Iran, the fear of losing patients, as doctors’ main source of income, for not prescribing antibiotics can sometimes override their willingness to adhere to medical guidelines and ethics. In addition, providing antibiotics over the counter worsens self-medication through increasing public access to antibiotics without prescription and encourages patients to buy more antibiotics as there is no significant negative feedback from authorities for selling antibiotics without prescription.

### Rules and values

Different types of formal antibiotics related rules, regulations, and guidelines were identified including pharmaceutical and medical guidelines and protocols, WHO and international guidelines, laws of IRIMC, codes, and regulations of insurance companies, and codes of actions and regulations developed by MOH. Most of them were authorized by MOH and publicly accessible. However, it was identified that they were not always followed by the agents.

Interviewees who were clinical pharmacists argued that not all physicians easily adhered to evidence-based medicine or collaborate with clinical pharmacists to change their antibiotic prescriptions. Some defied pharmaceutical guidelines and dismiss them in favor of their own experiences. Clinical pharmacists believed that physicians’ different following rules behaviors may also be influenced by their prior interaction experience with pharmacists, as well as health authorities.

Prescription and use of antibiotics behaviors were governed by the agents’ perception of formal rules, which could be termed internalized rules. According to some participants, physicians and patients follow their internalized rules more obediently than central and formal regulations. Even some subsystems like hospitals have their internal official regulations and guidelines toward infectious disease, derived from formal regulations but adapted to their contextual properties. We observed that pharmaceutical protocols were performing well in some private and public hospitals and were accepted by clinical pharmacists and Infectious disease specialists. However, some hospitals dismissed them because they believed pharmaceutical protocols should be internally developed based on the internal context of hospitals or there should be a reasonable room for change and adaptation, which was not the case in their hospitals. However, the results of the study revealed that this self-organizational behavior of subsystems is not well regarded by regulatory bodies in Iran.

In addition to rules, organizational, professional, and individual values were also recognized as contributing to governing certain types of behavior. They included organizational, professional, and individual values which could change overtimes as well. For example, insurance companies have clear and well-established credit and blame mechanisms that aim to restrict physicians’ over-prescription of antibiotics. Most participants agreed that rational use of antibiotics also greatly matters to governmental authorities due to the significance of antimicrobial resistance, which partly explains the establishment of Rational Use of Drugs committees in departments of Food and Drug Administration and hospitals. Likewise, among RUD indicators, the antibiotics use standard has always been an important indicator for MOH. However controversial it seems, some respondents argued that the importance of RUD committees had declined for MOH, and antibiotics have lost their priority. For example, except in insurance companies, there are no credit or blame mechanisms in governmental organizations related to the rational use of antibiotics and many respondents believed that their efforts to rationalize antibiotics use were not appreciated as much as they deserved. Some argued that the reason for the failure of NCRUD in reducing irrational use of antibiotics was the lack of support of its organizational position by MOH. Although the NCRUD is a national entity within the organizational structure of MOH, some interviewees believed that MOH does not delegate all RUD authority to it and support its enforcement.

In addition to organizational and professional values, individual values also influence the patients’ and physicians’ behavior about antibiotics. For example, some interviewees stated that sometimes people including physicians may have enough knowledge about the importance of rational use of antibiotics but their knowledge did not translate into attitude and practice.

Some participants noted that the magnitude of the rational use of medicines varies from person to person among policymakers and healthcare managers. During some periods, it might have attracted considerable attention and, under a different person’s management, it might have been consigned to oblivion.

### Dependent, diverse and multifactorial behaviors different of agents

We classified four major behaviors in the antibiotics use system conducted by key agents, all influenced by their information, rules, values, resources, feedbacks, and interaction with other agents.

#### Physicians’ prescription behaviors

Variation in physicians’ prescription behavior was expressed by a wide range of participants. Generally, all participants believed that physicians’ prescription behavior is influenced by the quality of their interactions with patients. Patients’ satisfaction seemed to be more important in contexts where physicians compete for more patients and more income. Noting the effect of context, some FGD participants believed that physicians exhibited different behaviors in the public and private sectors. Additionally, some physicians argued that they were faced with time constraints in some settings and did not have enough time to explain and convince patients that they do not need antibiotics. However, some people in FGDs reported contrary experiences with physicians who had spent reasonable time visiting them despite a long queue of patients.

#### Pharmaceutical companies and pharmacies behaviors

Data analysis of information provided by this study indicated that the behaviors of pharmaceutical companies and community pharmacies depended on many factors beyond their knowledge, the healthcare system, or its rules. For example, their practices were highly dependent on their income and so the economic, political, and even the international communication conditions of the country. In recent years, some technical and international communication problems have impeded pharmaceutical export and many pharmaceutical companies have lost their niche market in the Middle East. As a result, they have produced antibiotics over domestic use such that their inventories overflow with antibiotics leading to more efforts to sell antibiotics. Besides external limitations, there have been some restrictions imposed by MOH and IFDA on the procurement of the active pharmaceutical ingredients of antibiotics and pharmaceutical pricing, which can in turn further compound the situation. These forces altogether have driven companies to adapt themselves to an unpleasant situation by enhancing antibiotic sales through promotional activities. It was reported by participants that pharmaceutical companies sometimes provided some products like antibiotics free of charge to pharmacies as part of their promotional activities, offering financial incentives for physicians to over-prescribe antibiotics. These promotional activities seemed to be the most pressing concern repeated by almost all policymakers and prescribers interviewed in this study. On the other hand, economic pressures encourage pharmacies more to deliver antibiotics over the counter to the public in order to increase their revenues. Although these marketing strategies have increased the total use of antibiotics, they can be understood as an adapting behavior of companies and pharmacies.

#### Policymaking and implementation behaviors

Several interviewees discussed that the behaviors of policymakers depended on their willingness to achieve short-term or long-term outcomes, their personal preferences, characteristics and responsibility, contextual constraints, and experiences. One of the main reasons for the tendency toward short-term outcomes was identified as high-speed policy makers’ turnover. Rather certain in the knowledge that they are not likely to hold to their coveted positions for too long, policymakers often set themselves short-term achievable goals, which will be touted as their legacies. Participants believed that the importance of irrational use of antibiotics varied among different managers and policymakers, reflected in their policies and programs. Variations could also be easily seen in preferences and perceptions among interviewees at the policymaking level. For example, some stated that the rational use of antibiotics must strictly be rule-based, while others opposed and dismissed this view as an unnecessarily harsh practice. In addition, some argued that resource constraints especially financial limitations usually put more pressure on policymakers to allocate required resources for programs aimed to improve the rational use of antibiotics. Apart from personal preferences, it was also found that occasional crises, the context, and limitations dictate certain behaviors and attitudes toward adherence to rules.

#### Patients’ antibiotics use behavior

Data analysis revealed that people’s antibiotics use behavior was determined by many factors such as socio-economic situation, their perception of and belief in physicians, their perception of self-medication, antibiotics benefits and hazards, and their medical history. For example, while, there is no need for antibiotics, some participants according to their individual experiences, believed in the efficacy of antibiotics, while others thought that their efficacy was not overweighed by their side effects.

## Discussion

Many years of research and experimental policymaking to improve the rational use of antibiotics in Iran have been of little avail and it is still a mystery why all endeavors have yielded in few positive outcomes. There is a rather yawning gap between evidence, health planning, and real practices in managing health problems, mirroring the challenging task of narrowing this gap [46]. Against this backdrop, this study is an attempt to offer fresh insight into the complexity of the issue of irrational use of antibiotics in Iran and an in-depth analysis of the possible causes and solutions, building on the premises of the complex adaptive system theory.

The main strength of this study is examining a wide spectrum of different key agents and trying to understand the behaviors of the antibiotics use system through the lens of complexity science.

Findings provided a detailed explanation of the complexity of the issue by describing the antibiotics use system in Iran as a Complex Adaptive Systems shaped by highly diverse agents (stakeholders) and their properties, interactions, and behaviors regarding antibiotics use. The complexity perspective acknowledges that the system and its agents self-organize themselves and their behaviors and adapt according to new conditions utilizing feedback. Hence, the final behavior of the system and its agents is an emergent phenomenon of many interdependent factors, not fully predictable. We saw that physicians’ prescription behavior emerges from their trade-off between various variables such as their knowledge, attitude, patients’ satisfaction, and financial profits, besides official rules or clinical guidelines.

Overprescription of antibiotics is profitable for many stakeholders such as pharmaceutical companies, pharmacies, and physicians. Physicians can ensure their patients’ satisfaction by overprescribing antibiotics and maybe financially rewarded by pharmaceutical companies. Therefore, in absence of rules to limit these interactions and existing patients’ demand for more antibiotics, the prescription and consumption would be encouraged and increased regardless of its health consequences. Also, the results revealed that the decisions and attitudes of policymakers and healthcare managers about antibiotics use are highly affected by multiple co-changing factors including their personal preferences and priorities, interaction with other agents, external pressure, and the context in which they are practicing, not simply by evidence. Any intervention in any part of this system may bring about unexpected outcomes and, therefore, there should be some room for self-organizing and emergent behaviors of agents in order not to be shocked in the face of unexpected behaviors. Similarly, patients’ behavior regarding antibiotics depends on their past experiences of antibiotics use, their knowledge and attitude, and how fast they expect to recover regardless of their physicians’ advice. Such emergent behaviors toward a drug policy had been observed in other counties [29].

It was also observed that different agents in the system might pursue conflicting goals and interests. For example, while MOH and IFDA try to regulate the system, rationalize antibiotics use and reduce healthcare costs, pharmaceutical companies seek to gain more profit by offering financial incentives to pharmacies in exchange for buying more antibiotics. No wonder, then, pharmacies are encouraged to sell more antibiotics to patients or ask physicians to prescribe more antibiotics. In other words, while selling more antibiotics can save a pharmacy or pharmaceutical company and produce significant profits, or prescribing more antibiotics means more patients’ and pharmacies’ satisfaction and so higher income for physicians, it increases healthcare costs and antibiotic resistance as a serious health problem. So, it seems that most stakeholders’ goal is to maximize their profits rather than to uphold rules or address patients’ needs. Without a shared goal and values and effective internalized and widely accepted rules to prevent incentives for and benefits of irrational prescription and consumption of antibiotics, there is little chance for rational antibiotics use. It can be argued that unless adequate solutions are offered to address these conflicting interests of different agents in the system, the idea of rational antibiotics use will remain a far-fetched notion as ever.

Another key finding of this study was the interdependency of elements of the system which should be taken into account in understanding the problem and solution generation. It provided insight o how dependent behaviors of different agents of the system co-evolve by a change in the context of the systems or its agents can be. It needs to be understood that how financial incentives of some stakeholders or patients’ insistence to receive more broad spectrum antibiotics, might significantly influence the behavior and attitude of other stakeholders and finally lead to more prescription and consumption behavior. In view of this interdependency, in the network structure of the system, introducing change at any level or in any part of the system, may bring about unexpected outcomes in other components of the system and its final behavior. The key question is the leverage point of the system, the agents, or levels with the highest potential to improve the overall behavior of the system swiftly and inexpensively.

AS argued by Keshavarz et al., social CASs are more complex than biological or artificial complex adaptive systems as the rules might not be followed by the agents of the system [27], especially if there is no serious or immediate feedback or consequence for not following the rules. Our study showed that, besides the compromised mutual trust between health authorities and other agents, ineffective implementation and central supervision of rules have encouraged autonomous action often against policies and rules. Such behaviors can be seen in the behaviors of pharmaceutical companies, pharmacies, and hospitals. Despite many limiting regulations about pharmaceutical promotional activities and the prohibition of delivering antibiotics over the counter at pharmacies, antibiotic sales at pharmacies have been on the rise. Hospitals disobeyed some pharmaceutical protocols and guidelines dismissing them as incompatible with the pattern of antibiotic resistance in their hospitals. This study suggests that clinical guidelines and rules regarding the rational use of antibiotics are more likely to be followed by stakeholders if the rules are developed participatory, involving all stakeholders especially pharmacies and pharmaceutical companies, and academic associations, implemented locally and supervised continuously and effectively.

Another interaction that needs modification is the physicians’ and patients’ interaction. During a non-professional and insufficient interaction between physicians and patients, mutual trust is damaged and information is imparted to patients in a defective way. Essential information related to antibiotics use is not usually transferred to patients by physicians and pharmacists. Therefore, patients are not educated properly about the importance of rational use of antibiotics and consequently, their adherence to antibiotics therapy is decreased. An Improved physician-patient relationship may break down this reinforcing loop and help patients to adapt to interventions.

This study showed that how insufficient and non-systematic interactions between agents prevent good quality and timely information flow on antibiotics use. Improving the information flow in the rational use of antibiotics in Iran calls for more serious attention and should be achieved through the recovery of disrupted interactions, development of required infrastructures, and optimal use of the existing potentials.

In view of how the financial relationship between physicians and patients, on one hand, and between physicians and pharmacies and pharmaceutical companies, on the other hand, create conflicts of interest, these interactions need to be governed by a different structure and regulation. In other words, some interactions need to be banned or monitored by developing new rules and regulations. For example, promotional activities of pharmaceutical companies and their interactions with pharmacies and physicians need to be more regulated. In addition, physicians can be banned from cultivating any direct or indirect financial relationships with pharmacies and pharmaceutical companies. Negative impacts of the relationship between physicians and the pharmaceutical industry such as unethical irrational prescription behavior, unethical attitude, and malpractice in patient management have been demonstrated in previous studies [47, 48]. It also seems that the direct financial relationship between patients and physicians needs to be changed as it influences physicians’ behavior. The fee-for-service payment system has been substituted for new and modified payment models in many countries.

## Conclusion

Understanding the irrational use of antibiotics from a holistic perspective offered by the CAS observatory tool leads us to new directions and to generate solutions. To this end, by describing the irrational use of antibiotics through a CAS lens, this paper untangles the problem and provides a meaningful contribution to the literature. A substantial insight of this study would be noticing the complex nature of antibiotics irrational use problem and better managing unexpected consequences of any policy and program to address that.

The findings of the study suggest that the antibiotics use system in Iran needs re-engineering by strengthening some links between stakeholders, weakening or cutting some links, improving up-to-date antibiotics related information flow, and revising rules and values of the system; all these should be done, while taking into account the interdependencies of financial motivations, patients’ demands, professional knowledge and ethics, and autonomous and self-organized behaviors of agents.

It is our sincere hope that this study serves as a stepping stone and an incentive for like-minded researchers to further expand its scope and apply the principles of complexity science into other relevant areas of medicine use and prescription. Developing a dynamic model for the irrational use of antibiotics and CAS observatory studies can be some potential areas for future studies.

## Data Availability

All data generated or analyzed during this study are included in this published article.

## Abbreviations

CAS: Complex Adaptive Systems
NCRUD: National Committee on Rational Use of Drugs
WHO: World Health Organization
FGD: Focus Group Discussion
MHO: Ministry of Health
IFDA: Iran Food and Drug Administration
RUD: Rational Use of Drug
IRIMC: Islamic Republic of Iran Medical Council
NGO: Non-Governmental Organization
